# Tides of Change—Barriers and Facilitators to Beach Accessibility for Older People and People with Disability: An Australian Community Survey

**DOI:** 10.3390/ijerph20095651

**Published:** 2023-04-26

**Authors:** Sasha Job, Luke Heales, Steven Obst

**Affiliations:** 1 School of Health, Medical and Applied Sciences, College of Health Sciences, Central Queensland University, Bundaberg, QLD 4670, Australia; 2 School of Health, Medical and Applied Sciences, College of Health Sciences, Central Queensland University, Rockhampton, QLD 4701, Australia

**Keywords:** beach, blue space, nature, access, accessibility, disability, health, barriers, facilitators

## Abstract

The beach is Australia’s most popular recreational destination with participation in beach-based activities associated with a wide range of health and wellbeing benefits. Unfortunately, access to beach environments is not possible for many older people and people with a disability. The purpose of this study was to investigate the barriers and facilitators of beach accessibility using a framework that recognises the complex interconnections between blue space, accessibility, physical activity, and health and wellbeing. A 39-item anonymous online cross-sectional survey was developed and administered to explore the perspectives of older people and people with a disability regarding beach accessibility. In total, 350 people completed the survey (69% female, age range 2–90 years (mean = 52)). Disability was reported by 88% of respondents, with 77% requiring a community mobility aid. Two-thirds (68%) of respondents were unable to visit the beach as often as they wanted, with 45% unable to visit at all. The most frequently reported barriers to beach access included difficulty moving on soft sand (87%), no specialised mobility equipment (75%), and inaccessible lead-up pathways (81%). If beach access was improved, respondents reported they would visit the beach more often (85%), for longer (83%), and have an improved experience (91%). The most frequently reported facilitators to beach access were the presence of accessible lead-up pathways (90%), sand walkways (89%), and parking (87%). Older people and people with disability have limited beach access, primarily due to a lack of accessible equipment, excluding them from the wide range of health benefits associated with visiting the beach.

## 1. Introduction 

The proportion of the Australian population with a disability is increasing, largely due to an ageing population [1], placing pressure on disability and aged care service systems [1]. Because of this, novel strategies that address the key health inequalities between people with and without disability are needed [1,2,3,4,5,6]. Secondary to physical, attitudinal, and system-level barriers, people with disability face unique challenges accessing outdoor environments. As a result, people with disabiltiy are not afforded an equal opportunity to participate in health-promoting behaviours available in outdoor environments such as the beach and other blue space environments (e.g., lakes, rivers, and streams) [2,4]. 

The blue space literature is less established than it is for green space (e.g., wilderness, parks, and forests) [7]; however, there is emerging evidence that blue spaces are more widely used for health and wellbeing purposes [8,9] and may offer greater health benefits than green spaces [10,11,12,13]. Compared to green space, people who access blue space feel happier [10] and less stressed [13], and are more likely to participate in positive social interactions (e.g., family, friends) [12] and meet national physical activity guidelines [11]. Unfortunately, however, current blue space research is largely limited to the general population [14] so there is a need to explore how blue space environments can be used to promote health and wellbeing for people with unique accessibility needs (e.g., older people, people with disability). A recent systematic review including 33 studies examined the health and wellbeing impacts of blue space interventions for people with a defined therapeutic need [15]. Although the findings appear positive for the inclusion of blue space interventions for people with disability, only four studies included participants aged 65 years or over and only four studies reported outcomes for physical disability [15]. This review further highlights the need for research that considers the accessibility and impact of blue space environments within a framework that recognises the multidimensional nature of function.

The BlueABILITY model [16] integrates the International Classification of Functioning, Disability, and Health (ICF) [17] and the Physical Activity for People with a Disability (PAD) model [18] with White et al.’s [19] conceptual framework for Blue Space Health and Wellbeing. This model depicts the relationship between blue space, disability, and functioning, whilst recognising the diversity of how disabilities are individually experienced and the role of contextual (i.e., personal and environmental) factors as either barriers or facilitators to a person’s functioning. The BlueABILITY model also demonstrates how the model may be contextualised to beach accessibility [16]. Given that contemporary disability research has supported the need for generating knowledge of barriers and facilitators that is contextualised [20], this model was selected as our framework for investigating the barriers and facilitators to beach accessibility. 

The beach is Australia’s most popular recreational destination [21]. The recreational value of the beach is widely reported in terms of its immense economic [22,23], social [21,23], and environmental [22,23] significance; however, the lack of blue space health research in Australia highlights the potential oversight of the true value of the beach. Research shows that visiting the beach is associated with enjoyment, relaxation, and social interaction, as well as improved physiological, psychological, and social health and wellbeing [12,24,25,26,27,28]. Beaches also provide a sense of place and offer participation opportunities for activities that promote and improve wellbeing [23]. 

Unfortunately, a survey of 385 coastal residents from South-Eastern Australia indicates that 21.8% of Australians are unable to participate in their preferred beach activities, with 15.8% citing disability and/or old age as the primary barrier to participation [21]. Whilst this general population research highlights the inequity of beach access for older Australians and those with disability, very little is known about what specific factors influence their ability to go (or not go) to the beach. Although some studies suggest challenging terrains (e.g., sand, water), inappropriate access points, and inaccessible facilities (e.g., parking, amenities) are important [29,30], more research is needed to identify which factors are most influential and for which subgroups of people with disability (e.g., older versus younger, mobility versus non-mobility disability). Further, despite the incidence of disability increasing with age [3,31] and the known association between community environmental factors and disability in older adults [32,33,34], no research has explored beach accessibility from the perspective of older people with and without disability. 

Blue space research is emerging as a key priority area to address accessibility inequalities between people with different functional abilities. For example, a Spanish case study on beach accessibility outlined that the key elements of an accessible beach include both physical equipment and services to support beach use [35]. This study provided a blueprint for accessible beach design; however, it did not clearly present the barriers to beach access from the perspective of the individual. Further, as this research related to tourism, it did not consider the impact of beach access on health and wellbeing. A recent Australian study investigated beach accessibility using a social relational model of disability through the lived experiences of people with disability participating in beach sports [30]. The study highlighted that beach access for people with disability was dependent on services and assistance to support beach use [30], but did not outline the facilitators to beach access. Further, the perspectives of people with disability accessing the beach for reasons other than beach-based sports were not considered. Taken together, there remains a clear need for research that comprehensively considers the design (i.e., accessibility barriers and facilitators), impact (e.g., access rates), and community experiences of accessible beaches from the perspective of people with identified accessibility needs within a framework that recognises the multidimensional nature of blue space accessibility [16]. 

Therefore, the primary aim of this study was to characterise the barriers and facilitators to beach access for Australians who identify as having a disability and/or are of older age (>65 years) using the BlueABILITY framework [16] to describe the main environmental and personal factors that influence beach accessibility. The secondary aim was to determine whether these barriers and facilitators differed based on their age and disability or mobility status. 

## 2. Materials and Methods 

### 2.1. Study Design

The study was a cross-sectional anonymous online survey of Australian people who identified as a person with a disability and/or were of older age (≥65 years). To ensure transparency and reproducibility of the survey, it was designed according to recent recommendations and recent practices in Australian physiotherapy research [36,37,38,39,40,41]. The study was approved by the Central Queensland University Human Research Ethics Committee (approval number 0000022411). 

### 2.2. Recruitment and Participants

Participants were recruited via three methods: (1) flyer advertisement, (2) social media, and (3) mainstream media. The flyer advertisement was displayed on noticeboards in healthcare settings (e.g., physiotherapy private practices), education facilities (e.g., universities), and community disability organisations. In addition, a flyer containing a link to the survey was distributed via email and social media (Facebook, Twitter, LinkedIn) in April 2021 to community-based disability, aged care, health, and recreational services as well as professional networks (e.g., Accessible Beaches Australia, National Disability Insurance Scheme (NDIS) provider networks). The CQUniversity marketing directorate disseminated the research to mainstream media. A second round of social and mainstream media advertising occurred four weeks after the initial flyer distribution [42]. The survey was open from April 2021 to October 2022.

Participants were eligible to complete the survey if they currently accessed or wanted to access patrolled beaches throughout Australia and self-identified as: (1) an older person (>65 years) and/or (2) a person living with a disability. For individuals aged under 18 years, the survey requested that a parent/guardian answered the questions acting on their behalf. For individuals unable to complete the survey independently, the survey was able to be completed by a parent/guardian, carer, or service provider on their behalf. Participants were excluded if they did not meet the inclusion criteria using pre-screening questions. Participation was voluntary, and all respondents provided informed consent prior to survey completion.

### 2.3. Survey Instrument 

The survey was built and administered using Qualtrics (Qualtrics, Provo, UT, USA). This was the first study to explore beach accessibility from the perspective of older people and people with disabiltiy in the healthcare context and thus no existing questionnaires were identified that were appropriate to address the research questions. Therefore, a purpose-designed questionnaire informed by the literature was developed and peer-reviewed by a team of physiotherapists (n = 3) and occupational therapists (n = 2) with experience in disability and accessibility research and clinical practice. The BlueABILITY model [16] was used as a framework for the survey, with questions designed to address all levels of the model (i.e., functioning factors, contextual factors, blue space exposure, health and wellbeing outcomes, and interventions). Survey questions were designed to obtain data on the following major themes: (1) patterns and preferences of beach use and accessibility, (2) perceptions and benefits of beach use and accessibility, and (3) barriers and facilitators to beach use and accessibility. For the purposes of the current paper, only data pertaining to the final theme will be discussed. Data pertaining to the remaining themes will be presented in future papers. 

The 39-item questionnaire (see Supplementary Material S1) was structured into four sections: (1) demographics (12 questions), (2) physical activity participation (7 questions), (3) patterns and preferences of beach use (12 questions), and (4) beach accessibility (8 questions). For the beach accessibility section specifically, questions related to general accessibility (5 questions on perceptions, motivations, impacts), barriers to beach access (2 questions), and facilitators (1 question that included specialised equipment images). 

Questions included a combination of categorical, ordinal (5-point Likert scale), and open response data. Categorial questions were predominantly used for the demographic data (e.g., gender, identification as a person with a disability and/or of older age). Ordinal data were obtained for agreement with accessibility statements (e.g., barriers and facilitators (strongly disagree, disagree, neither agree or disagree, agree, strongly agree) and health-related questions (e.g., self-reported health (poor, fair, good, very good, excellent)). Open-ended questions were used to capture deeper and/or new insights into lived experiences and impacts of beach accessibility (e.g., influence of mobility limitations on beach experience). Images (e.g., specialised beach equipment) were included to facilitate the interpretation of questions when required (e.g., facilitators). Prior to distribution, the full survey was piloted (n = 10) to assess question structure, distribution, and data quality [43]. No changes were made to the survey following the pilot. As participation incentives were not offered for this survey, the risk of bots was anticipated to be minimal; however, screening of survey completion location, time, and speed was used to check for potential bot responses [44].

### 2.4. Sample Size Calculation 

Based on Australian population data [3,5] for people with disability (~4.4 million) and people aged 65 years and over (4.2 million), we estimated a minimum target sample size of 349 when using a 5.5% margin of error, 5% response distribution, and 95% confidence limit [45]. 

### 2.5. Analysis 

For the current study, only quantitative data pertaining to participant demographics and perceived barriers and facilitators to beach access are presented and discussed. Demographic and survey question data for all participants are summarised using descriptive statistics (e.g., frequency, mean, SD). Group differences in response data for the barrier and facilitator questions based on the following age and disability subgroups (group 1: <18 years with a disability; group 2: 18–64 years with a disability; group 3: ≥65 years with a disability; and group 4: ≥65 years without disability) were compared using Kruskal–Wallis H tests, with post hoc Mann–Whitney U tests as required. Group differences in response data for the barrier and facilitator questions were also compared between people with mobility and non-mobility disabiltiy using Mann–Whitney U tests. Statistical significance was set at an alpha level of 0.05. Effect sizes for the Mann–Whitney U tests were calculated using the z-score using the equation $r=\frac{z}{\sqrt{n}}$, and reported as small (<0.3), moderate (0.3–0.49), or large (≥0.5) [46]. Barriers and facilitators determined to have a high level of agreement (agree or strongly agree) by the highest proportions of respondents were considered to indicate the greatest importance in the context of accessibility planning and initiatives. 

## 3. Results

### 3.1. Respondent Characteristics 

A total of 406 people completed the eligibility screening questions, and of these, 350 were deemed eligible to participate. Completion rates were as follows: 100% (n = 218), 80–99% (n = 29), 50–79% (n = 31), and 30–49% (n = 72). For the current study all response data, including partial/incomplete responses, were included in the analysis. No responses were excluded based on screening procedures for potential bots. 

A summary of respondent demographics for all participants (n = 350) is presented in Table 1. The average age of respondents was 52.0 years (SD = 19.7), and most were female (n = 239, 68.3%). Older individuals (≥65 years of age) accounted for 32.0% of respondents. Most respondents (87.7%) reported having a disability. Forty-three (12.3%) respondents identified as an older person without identifying as a person with a disability; however, 25.6% of these respondents reported that they required assistance with activities of daily living or mobility, and/or required the use of a mobility aid. The sample sizes and ages of the age and disability status subgroups were as follows: group 1 (n = 23, 7%; mean (SD) age = 10.9 (3.6) years), group 2 (n = 203, 58%; mean (SD) age = 44.8 (12.2) years), group 3 (n = 81, 23%; mean (SD) age = 71.6 (9.7) years), and group 4 (n = 43, 12%; mean (SD) age = 70.9 (5.9) years)). The sample sizes and ages of the mobility subgroups were as follows: mobility disability (n = 101, 66%; mean (SD) age = 56.8 (17.7) years) and non-mobility disability (n = 53, 34%; mean (SD) age = 49.0 (13.4) years). 

**Figure 1:**
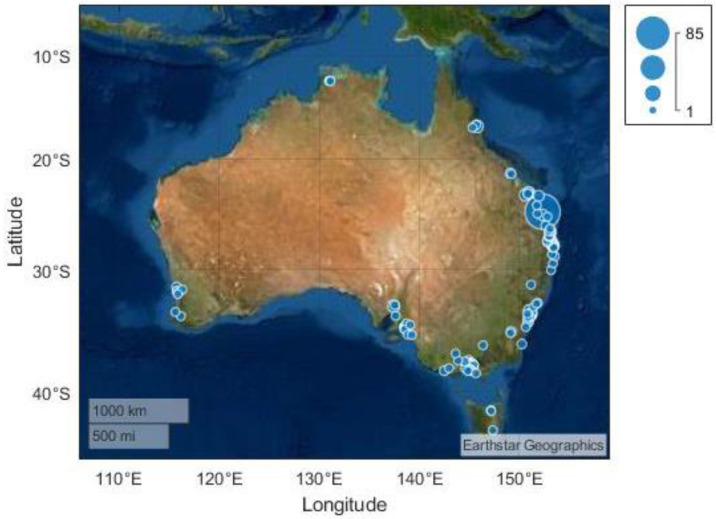
Location of respondents across Australia. Postcode geography is displayed using dot circumference to indicate the number of respondents in each area.

Most respondents (64.9%) rated their health status as good to excellent. Approximately half of the respondents reported they required assistance with self-care (49.1%) and/or mobility (53.8%), with most requiring a mobility aid at home (66.9%) and within the community (76.9%). The most frequently used mobility devices were wheelchairs (manual—39.3% and power—23.7%), wheeled walking frames (30.8%), and walking sticks (23.1%). Three-quarters of respondents (74.6%) lived within 30 km of a patrolled beach, with 43.1% living within 10km. Respondents were mostly located along the east coast of Australia, with a large proportion based within the Wide Bay region of Queensland (Figure 1).

### 3.2. Barriers to Beach Access 

**Figure 2:**
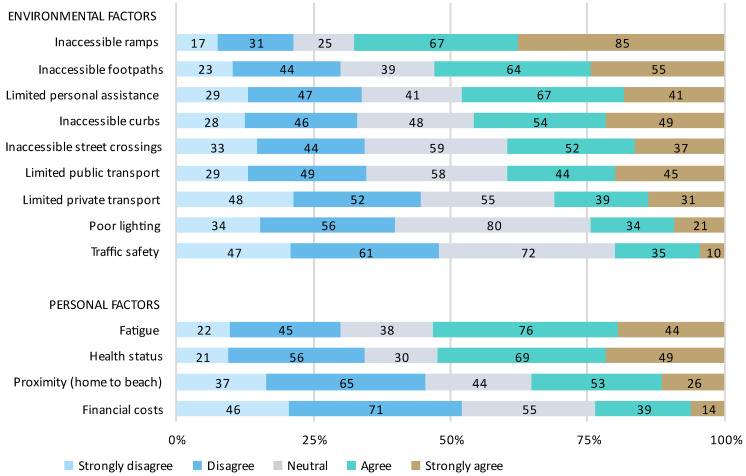
Barriers to travelling to the beach. Percentage of respondents who strongly disagree, disagree, are neutral, agree, or strongly agree that each environmental or personal factor is a barrier is presented in order of agreeance.

**Figure 3:**
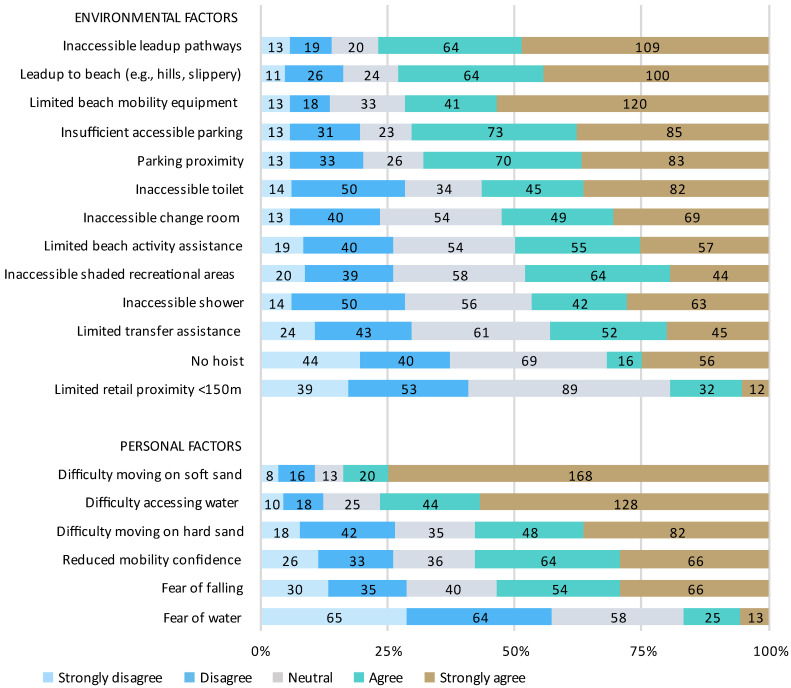
Barriers to accessing the beach and surrounds. Percentage of respondents who strongly disagree, disagree, are neutral, agree, or strongly agree that each environmental or personal factor is a barrier is presented in order of agreeance.

Almost half of the respondents (44.8%) were unable to visit the beach at all. Of those that were able to visit the beach, 68.0% were unable to visit as often as they would like to, and 63.6% were unable to spend as much time at the beach as they would like. A summary of the barriers to beach access is presented in Figure 2 and Figure 3. The most frequently reported environmental barriers to travelling to the beach (Figure 2) were inaccessible ramps (67.6%) and footpaths (52.9%) and limited personal assistance (48.0%), with the most frequently reported personal barriers being personal fatigue (53.3%) and health status (52.4%). The most frequently reported environmental barriers to accessing the beach (Figure 3) included inaccessible lead-up pathways (76.9%), no/limited specialised beach mobility equipment (71.6%), and inaccessible or limited parking options (70.2%). Inaccessible toilets (56.4%) and change rooms (52.4%) were also commonly identified barriers. The most frequently reported personal barriers were difficulty moving on soft (83.6%) and hard sand (57.8%), difficulty accessing the water (76.4%), reduced confidence with outdoor mobility (57.8%), and fear of falling (53.3%). 

The results of the subgroup analyses for the barriers to beach access based on age and disability status are presented in Table 2. There was a main effect of the group for 18 out of the 19 reported barriers. Post hoc Mann–Whitney U tests revealed that the barriers for people with disabiltiy of any age (groups 1–3) were consistently greater than those for older people without disabiltiy (group 4). These differences were most evident for questions relating to sand and water mobility, and the need for specialised beach mobility equipment, where effect size estimates ranged from 0.35 (moderate) to 0.80 (large). In contrast, there were only a few small differences in the barriers to beach access identified between groups 2, 3, and 4, most of which related to the accessibility of amenities (e.g., shower, toilet, and change room).

The results of the subgroup analyses for the barriers to beach access based on disability and mobility status are presented in Supplementary Material S2. There was an effect of the group for 4 out of the 19 reported barriers. Differences were evident for questions relating to accessible facilities (toilet, shower, and change room) and hoist availability; however, effect size estimates were small and ranged from 0.18 to 0.26.

### 3.3. Facilitators to Beach Access

**Figure 4:**
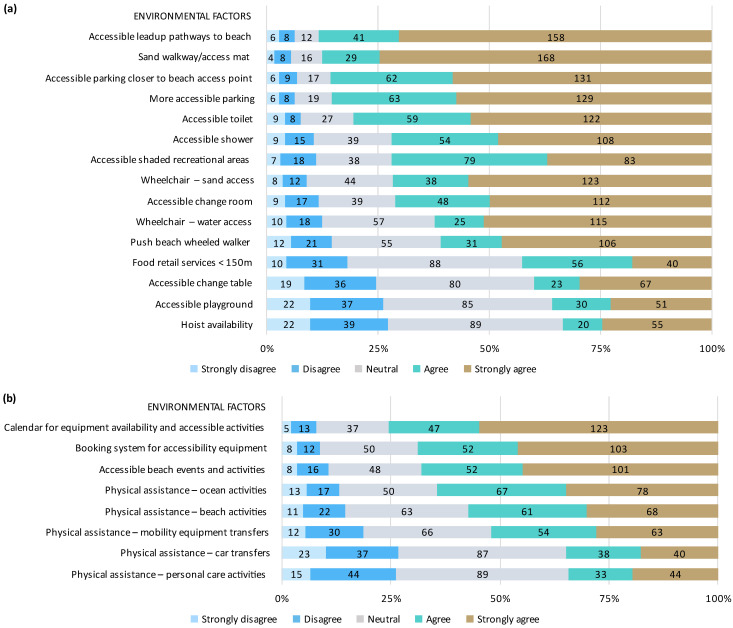
Facilitators to accessing the beach and surrounds. (**a**) Physical access to the beach and surrounds, (**b**) services to support beach use. Percentage of respondents who strongly disagree, disagree, are neutral, agree, or strongly agree that each environmental factor is a facilitator is presented in order of agreeance.

Most respondents reported they would visit the beach more often (85.4%), for longer (83.0%), and would have a better experience (90.7%) if access was improved. A summary of the facilitators to beach access is presented in Figure 4. The most frequently reported facilitators to physical beach access were accessible lead-up pathways and ramps to the beach (90.0%), sand walkways or access mats (89.1%), and accessible parking (86.9%) close to the beach access point (87.3%). The most frequently reported services to facilitate beach use were a calendar of equipment availability and accessible events/activities (76.9%), a booking system for accessibility equipment (70.1%), and physical assistance to support both ocean (65.6%) and beach (58.4%) activities. 

The results of the subgroup analyses for the facilitators to beach access (physical equipment and support services) based on age and disability status are presented in Table 3. There was a main effect of the group for 19 out of the 23 reported facilitators. Post hoc Mann–Whitney U tests revealed that the facilitators for people with disabilities of any age (groups 1–3) were consistently greater than those for older people without disabilities (group 4). These differences were most evident between group 4 and group 1, and group 4 and group 2, wherein small–large effect sizes (r = <0.3–>0.5) were found for 19 out of 23 and 23 out of 23 facilitators, respectively. The fewest differences were found between group 2 and group 3, and group 2 and group 1, wherein only 2 and 8 of the 23 facilitators, respectively, were significantly different.

The results of the subgroup analyses for the facilitators to beach access based on disability and mobility status are presented in Supplementary Material S3. There was a main effect of the group for 4 out of the 23 reported facilitators. Differences were evident for questions relating to a wheelchair for water access, accessible facilities (toilet and change room), and hoist availability, where effect size estimates were small and ranged from 0.16 to 0.17.

## 4. Discussion

This study investigated beach accessibility barriers and facilitators from the perspective of older people and people with disability. The most frequently agreed-upon barriers to beach access were inaccessible lead-up pathways to the beach, difficulty moving on soft sand, and difficulty accessing the water. Whilst a large proportion of our respondents were unable to visit the beach at all, most indicated they would visit the beach more often, for more time, and have an improved experience if beach access was improved. The most frequently reported facilitators to beach access related to both physical access (e.g., sand access mats) and services to support beach use (e.g., calendar of equipment availability and accessible events and activities). Importantly, we found clear differences in response data based on age and disability status. Older respondents without self-reported disability had fewer and less important barriers and facilitators to beach access compared to people with disabiltiy, regardless of their age group. Similarly, regardless of their age, people with disabiltiy reported similar barriers and facilitators to beach access. This study demonstrates the diversity of beach experiences and highlights the importance of person-centred co-design for inclusion in beach environments. 

At a community level, increased demand for accessible beaches has been reported [35,47]. Despite this, our data show that access to the beach is not equal for all Australians. The reported rates of beach use in the current study are vastly different to those reported for the general Australian population. In the current study, nearly half of the respondents (45%) never visited their local beach compared to the 1% reported for the general Australian population [21]. Further, approximately two-thirds of our respondents reported that they were unable to visit the beach as often (68%) or for as long (64%) as they would like to because of poor accessibility. In comparison, only 22% of the general population reported they were unable to participate in their preferred beach activities [21]. Our results clearly highlight the accessibility gap between older people and people with disabiltiy and those of the general population for enjoying the multiple health and wellbeing benefits that are associated with participation in recreational activities in natural environments [28,48,49]. As Australia is a signatory of the United Nations Convention on the Rights of People with Disabilities [6], it is essential that appropriate measures be taken to ensure people with disability can participate in their preferred beach activities and enjoy the highest attainable standard of health and wellbeing. 

Our study showed that older people and people with disabiltiy report numerous barriers and facilitators when travelling to and accessing the beach. Whilst the absence of a barrier is not necessarily a facilitator for societal participation [50], in most cases we observed similar response data for related (but opposing) barriers and facilitators to beach access. Previous work has also identified facilitators and barriers to often be an inverse of each other [51]; hence, facilitators and barriers will be discussed together in the context of environmental and personal factors, and in accordance with the BlueABILITY model proposed by Job et al. [16]. 

### 4.1. Barriers and Facilitators to Travelling to the Beach

Albeit less important than barriers to accessing the beach and its surroundings, our respondents identified environmental and personal barriers to travelling to the beach. Although no existing evidence for barriers specific to beach travel was identified, this finding aligns with daily travel behaviour research that has found that the diverse travel needs and behaviours of people with disabiltiy are not well considered in community travel demand modelling and planning practice [52]. The most frequently reported barriers to travel in our study were inaccessible ramps and footpaths. Poor accessibility of the built environment has been shown to change, delay, and cancel travel for people with disability [52,53]. Together, these findings suggest that the impact of the built environment on travelling to the beach may be significant, and thus further investigation into travel demand modelling and planning practices is warranted. 

Personal factors also affect travel behaviour [52], with the most frequently reported personal barrier to beach travel in our study being fatigue. Generally, people with disability encounter barriers to their community transportation which results in them having increased travel time and travel costs (e.g., vehicle adaptation, specialised ride services) compared to people without disability [52]. In this context, extended travel time may exacerbate fatigue and lead to a reduced beach trip frequency and less time being spent at the beach. Interestingly, financial costs were not identified as a barrier to beach travel in this study, which may reflect the prioritisation of government funding to support social and community participation as part of the Australian NDIS. While the investigation into the impacts and facilitators to community travel was outside the scope of this study, studies propose that the safety, inclusion, and connectivity of people with disabiltiy must be better considered in community transportation policies, practices, and guidelines [52]. Additional research is required to understand beach travel to better optimise the time older people and people with disability spend at the beach.

### 4.2. Barriers and Facilitators to Accessing the Beach and Surrounds 

Similar to previous beach accessibility studies [29,30], the most frequently agreed-upon barriers and facilitators to accessing the beach and surrounds were related to environmental factors, which are concerned with natural and human-made changes to the environment, products and technology, support, services, and systems and policies [17]. For example, our data highlight the importance of providing physical access to the beach and surrounding areas. Consistent with studies in recreation [30] and tourism [35] settings, we identified that the most frequently reported factors were related to accessible lead-up pathways and ramps to the beach, sand walkways or access mats, and the provision of specialised beach mobility equipment. This was not surprising given the known challenges that variable beach terrains (e.g., soft sand) pose for people with disabiltiy [54]. 

Factors related to parking and accessibility of beach precinct facilities (e.g., toilets) were also frequently identified. Consistent with previous studies [30,35], our respondents reported that more accessible parking located in closer proximity to the beach access point would facilitate beach access. Adapted facilities are essential to ensuring a level of comfort and hygiene for all beach users [54], thus to support equal access to beach environments, facilities must be designed according to universal accessibility standards [6,30,54]. 

In addition to physical access, the provision of services to support beach access will be required to improve beach accessibility. In our study, physical assistance was reported by respondents to be an important support service to facilitate beach access, with many identifying that assistance for both beach (sand) and ocean (water) activities would support their beach access. This finding is in alignment with previous research that suggests the elements of an accessible beach relate to both equipment and services to support beach use [30,35]. Consistent with our finding of accessible beach activities and events facilitating beach access, the availability of support services for adapted beach activities (e.g., surfing) has been shown to incentivise beach visits and participation in health-promoting physical activities [54]. 

A calendar of equipment availability and accessible events/activities as well as a booking system for accessibility equipment were identified by respondents in our study as a facilitator of beach access. In recognition of the higher effort of planning required to plan daily travel [52], it is essential that beach accessibility systems be implemented to support the ‘know before you go’ sentiment that is promoted through government initiatives (e.g., NDIS) and other community organisations (e.g., Spinal Life Australia). Australia’s coastline extends approximately 34,000 km [55], and thus in this context, it is clear that technology must play a central role in improving beach access by providing tools to enhance awareness and knowledge of beach access equipment and services [35]. We recommend the development of a comprehensive accessible beach visitor management system that complies with Web Content Accessibility Guidelines (WCAG) [56]. This system should provide an interactive accessible beach directory that serves as an accessible beach asset map with accessibility descriptions and ratings, 360° virtual tours, an equipment and service booking engine, and links to accessible beach activities and events. 

Our results indicate that if beach accessibility was improved, older people and people with disabiltiy would visit the beach more often, for more time, and have an improved beach experience. This is an important finding, as current research supports a dose-response to natural environment access that suggests greater exposure to green and blue spaces is associated with greater health benefits [24,57,58]. There is also evidence to suggest that natural environments may be ‘equigenic’, whereby the health benefits linked with access are the strongest among disadvantaged groups [59]. Natural environments are not equally accessed by all individuals, including people aged 65 years or over, and those excluded by economic disadvantage or disability [60,61]. Research has shown that although these inequities in access to the natural environment contribute to health inequalities, improving access to natural environments is associated with improved health and wellbeing [60]. For example, engaging and innovative visits to green spaces led by collaborative community partnerships (e.g., universities, schools, councils, healthcare providers) have demonstrated an increase in the use of green space by people typically excluded from the natural environment [60]. As such, people typically excluded from green space are more likely to be physically active and experience better health outcomes, which may reduce disability and socioeconomic-related inequalities in health [60]. 

We know that natural and adapted environments contribute to the exclusivity of the beach [54]; however, we identified that personal factors also have an important influence on beach access. For example, respondents reported mobility difficulty (e.g., difficulty moving on soft sand and accessing the water) and concerns such as fear of falling, which have been shown to restrict participation in physical and social activities [62]. Further, limited visibility of disabilities in beach environments reinforces social exclusion and discriminatory attitudes towards people with disabiltiy [30]. When taken together with the diversity of the individual disability experience, it is therefore essential that measures to improve beach accessibility recognise the complex interconnections between beach access and all contextual (i.e., environmental and personal) factors [16,17]. 

### 4.3. Beach Access for Older People and People with Disability (Mobility and Non-Mobility) 

The results of the group comparisons showed that there were statistically significant differences in almost all barriers and facilitators to beach access for respondents grouped according to their age and disability status. For example, an accessible change room is more of a factor for younger people with disability compared to older people with and without disability, but parking proximity to the beach access point is more of a factor for adults with disability compared to younger people with disability and older people with and without disability. Similarly, a push beach walker is more of a factor for older people with disability compared to younger people with disability and older people without disability. These differences in the barriers and facilitators to beach accessibility likely reflect the multifaceted nature of the disability and significant diversity in the lived experiences of people with disability. 

Interestingly, there was no consistent evidence for us to draw firm conclusions that differences in the barriers and facilitators to beach access exist between people with mobility and non-mobility disability. Despite no difference in age, significant differences between these groups were only found for a small number of factors related specifically to accessible facilities, wheelchair access, and hoist availability, and the effect sizes for these factors were considered small. These findings likely reflect the multi-dimensional nature of disability and the challenges associated with subgroup analysis of survey data based on broad classifiers. To further understand the impact of disability type on beach access, further research involving larger sample populations and qualitative methodologies will be required. 

Our findings offer insights into the differences some contextual (i.e., environmental and personal) factors may have on disability inclusion in beach environments. When taken together with existing research that has examined social, cultural, and gender differences in beach environments [30,54,63,64], it is clear that equal opportunity is an ideal not always realised in beach settings. The diversity of disability experiences has not always been well represented in Australian disability policy [65]; however, the differences in the barriers and facilitators to beach access between people of different disability statuses and ages demonstrate that the process of co-design is essential to improving beach access for all Australians. Further research and community engagement in decision-making to improve the accessibility of beach environments are required. 

### 4.4. Limitations and Future Directions 

Although this study is the first Australian survey to investigate the barriers and facilitators to beach access from the perspective of older people and people with disability, some limitations should be noted. Capturing the diverse experiences of people with disability in a reporting context is challenging. We recognise that people with disability are not a homogeneous group and that our study population is diverse, encompassing people with varying types and levels of disability across all socioeconomic and demographic groups. Despite recruiting participants across a large geographical area that represents all Australian states and territories, the sample was largely comprised of people residing along the east coast of Australia which may limit generalisability. Some respondents did not complete the full survey, which may have been because of the time required for participation. Additionally, barriers and facilitators were self-reported, which may be subject to biases such as recall and social desirability. Further, although the barriers and facilitators presented in this paper provide important knowledge on the contextual factors related to beach accessibility, they are presented from a quantitative perspective. Thus, despite being contextualised to the beach using the BlueABILITY model [16], they are isolated from the context in which they were experienced. Further qualitative investigation is required to contextualise individual beach experiences. 

Despite the increased community-level demand for accessible beaches [35,47], our data show that access to the beach is not equal for all Australians and there remain significant deficiencies in beach access, equipment, and services in most Australian regions. To better understand the impacts of exclusion from beach environments, further investigation into the health benefits (physical, mental, and social) of beach access including the examination of how beach access (e.g., frequency of visiting the beach) is associated with health status (e.g., self-reported health, physical activity participation) is required. Research should also be conducted to explore the perspectives of other key accessibility stakeholders (e.g., health professionals, surf lifesaving) and to evaluate the effectiveness of implemented beach access initiatives in overcoming identified barriers and supporting the increased use of the beach setting to promote health and wellbeing for people with disability. 

## 5. Conclusions

Our findings demonstrate that older people and people with disability face many barriers to accessing beach environments. Overcoming the identified barriers to beach access may be associated with increased time spent at the beach and therefore improved health outcomes. Elements of an accessible beach should include the consideration of the built environment (e.g., accessible parking, accessible lead-up pathways and ramps to the beach), physical equipment (sand walkway or access mats, specialised beach mobility equipment), and services to support beach use (e.g., beach accessibility calendars and booking systems, physical assistance for beach and ocean activities). It is recommended that such measures are initially employed at patrolled beaches throughout Australia given their widespread localities, proximity to people with disability, and practical considerations (e.g., storage of equipment and existing accessible infrastructure and facilities). The diversity in barriers and facilitators to beach access highlights the importance of further research and community-level decision-making for beach accessibility initiatives. 

## Figures and Tables

**Table 1:** Respondent characteristics. Data presented as n (%) unless otherwise stated.

Characteristic	Category	Data
Age (years) mean (SD)		52.0 (19.7) (range 2–90)
Gender	Female	239 (68.3%)
	Male	103 (29.4%)
	Non-binary	4 (1.1%)
	Prefer not to say	4 (1.1%)
Disability status *	Disability	185 (65.4%)
	Disability with mobility limitation	103 (29.4%)
	Older person (≥65 years)	112 (32.0%)
	Older person (≥65 years) with disability	66 (18.9%)
	Carer or service provider of person with disability	94 (26.9%)
Assistance required *	Self-care physical assistance	166 (49.1%)
	Mobility physical assistance	182 (53.8%)
	Home mobility aid	226 (66.9%)
	Community mobility aid	260 (76.9%)
Mobility aid *	Walking stick	78 (23.1%)
	Crutches	24 (7.1%)
	Walking frame—pickup	3 (0.9%)
	Walking frame—wheeled	104 (30.8%)
	Wheelchair—manual	133 (39.3%)
	Wheelchair—power	80 (23.7%)
	Scooter	31 (9.2%)
	Other	21 (6.2%)
Self-reported health	Excellent	30 (8.6%)
	Very Good	85 (24.3%)
	Good	112 (32.0%)
	Fair	88 (25.1%)
	Poor	35 (10.0%)

Note: * characteristic percentage does not sum to 100 as participants could select multiple options.

**Table 2:** Kruskal–Wallis H and Mann–Whitney U results for barriers to beach access for age and disability subgroups.

Barrier	Kruskal–Wallis H	Mann–Whitney U					
		Group 1 vs. Group 2	Group 1 vs. Group 3	Group 1 vs. Group 4	Group 2 vs. Group 3	Group 2 vs. Group 4	Group 3 vs. Group 4
Limited accessible parking	11.727 ***p* = 0.008**	1071.500; *p* = 0.506 MR Group 1 = 69.03 MR Group 2 = 75.82 r = −0.08	432.000; *p* = 0.893 MR Group 1 34.50 MR Group 3 33.82 *r* = −0.02	157.500; ***p* = 0.040** MR Group 1 27.75 MR Group 4 19.83 r = −0.31	2889.500; *p* = 0.277 MR Group 2 92.94 MR Group 3 83.97 r = −0.08	1080.000; ***p* =< 0.001** MR Group 2 84.76 MR Group 4 54.00 r = −0.27	459.500; ***p* = 0.023** MR Group 3 42.62 MR Group 4 31.02 r = −0.26
Parking proximity to beach access point	22.590 ***p* =< 0.001**	843.500; ***p* = 0.038** MR Group 1=56.36 MR Group 2=77.56 r = −0.17	416.000; *p* = 0.715 MR Group 1 32.61 MR Group 3 34.51 r = −0.04	167.500; *p* = 0.074 MR Group 1 27.19 MR Group 4 20.20 r = −0.27	2453.500; ***p* = 0.023** MR Group 2 95.58 MR Group 3 76.91 r = −0.17	868.500; ***p* =< 0.001** MR Group 2 86.37 MR Group 4 46.17 r = −0.35	457.000; ***p* = 0.022** MR Group 3 42.67 MR Group 4 30.93 r = −0.26
Physically challenging geography leading to beach	33.196 ***p* =< 0.001**	1121.000; *p* = 0.711 MR Group 1 = 71.78 MR Group 2 = 75.44 r = −0.05	385.000; *p* = 0.402 MR Group 1 37.11 MR Group 3 32.86 r = −0.10	92.500; ***p* =< 0.001** MR Group 1 31.36 MR Group 4 17.43 r = −0.53	2701.500; *p* = 0.077 MR Group 2 94.38 MR Group 3 80.13 r = −0.13	630.500; ***p* =< 0.001** MR Group 2 88.19 MR Group 4 37.33 r = −0.45	304.000; ***p* =< 0.001** MR Group 3 45.80 MR Group 4 25.26 r = −0.46
Inaccessible lead-up pathways to beach	25.514 ***p* =< 0.001**	1155.000; *p* = 0.877 MR Group 1 76.33 MR Group 2 74.82 r = −0.02	386.500; *p* = 0.404 MR Group 1 37.08 MR Group 3 32.89 r = −0.10	108.500; ***p* = 0.001** MR Group 1 30.47 MR Group 4 18.02 r = −0.48	2862.000; *p* = 0.220 MR Group 2 93.15 MR Group 3 83.41 r = −0.09	767.000; ***p* =< 0.001** MR Group 2 87.15 MR Group 4 42.41 r = −0.40	355.000; ***p* =< 0.001** MR Group 3 44.76 MR Group 4 27.15 r = −0.40
Inaccessible toilet	15.134 ***p* = 0.002**	1139.000; *p* = 0.807 MR Group 1 77.22 MR Group 2 74.69 r = −0.03	340.000; *p* = 0.137 MR Group 1 39.61 MR Group 3 31.94 r = −0.18	137.000; ***p* = 0.011** MR Group 1 28.89 MR Group 4 19.07 r = −0.38	2559.500; ***p* = 0.030** MR Group 2 95.46 MR Group 3 77.23 r = −0.16	1039.000; ***p* =< 0.001** MR Group 2 85.07 MR Group 4 52.48 r = −0.28	529.500; *p* = 0.139 MR Group 3 41.19 MR Group 4 33.19 r = −0.17
Inaccessible shower	14.308 ***p* = 0.003**	932.000; *p* = 0.136 MR Group 1 88.72 MR Group 2 73.11 r = −0.18	300.500; ***p* = 0.041** MR Group 1 41.81 MR Group 3 31.13 r = −0.25	101.000; ***p* =< 0.001** MR Group 1 30.89 MR Group 4 17.74 r = −0.50	2792.000; *p* = 0.167 MR Group 2 93.69 MR Group 3 81.98 r = −0.01	1110.500; ***p* = 0.002** MR Group 2 84.52 MR Group 4 55.13 r = −0.25	518.000; *p* = 0.109 MR Group 3 41.43 MR Group 4 33.19 r = −0.18
Inaccessible change room	22.282 ***p* =< 0.001**	784.500; ***p* = 0.017** MR Group 1 96.92 MR Group 2 71.99 r = −0.20	225.500; ***p* = 0.002** MR Group 1 45.97 MR Group 3 29.60 r = −0.39	71.000; ***p* =< 0.001** MR Group 1 32.56 MR Group 4 16.63 r = −0.61	2561.500; ***p* = 0.032** MR Group 2 95.45 MR Group 3 77.28 r = −0.16	1062.500; ***p* =< 0.001** MR Group 2 84.89 MR Group 4 53.35 r = −0.27	548.500; *p* = 0.207 MR Group 3 40.81 MR Group 4 34.31 r = −0.14
Difficulty moving—soft sand	65.742 ***p* = 0.011**	1032.000; *p* = 0.181 MR Group 1 83.17 MR Group 2 73.89 r = −0.16	364.000; *p* = 0.103 MR Group 1 38.28 MR Group 3 32.43 r = −0.20	48.000; ***p* =< 0.001** MR Group 1 33.83 MR Group 4 15.78 r = −0.72	3062.500; *p* = 0.489 MR Group 2 91.62 MR Group 3 87.50 r = −0.05	501.500; ***p* =< 0.001** MR Group 2 89.17 MR Group 4 32.57 r = −0.59	186.500; ***p* =< 0.001** MR Group 3 48.19 MR Group 4 20.91 r = −0.66
Difficulty moving—hard sand	22.891 ***p* =< 0.001**	1043.000; *p* = 0.408 MR Group 1 82.56 MR Group 2 73.96 r = −0.10	412.000; *p* = 0.665 MR Group 1 35.61 MR Group 3 33.41 r = −0.05	91.000; ***p* =< 0.001** MR Group 1 31.44 MR Group 4 17.37 r = −0.54	3071.500; *p* = 0.643 MR Group 2 89.45 MR Group 3 93.32 r = −0.03	833.00; ***p* =< 0.001** MR Group 2 86.64 MR Group 4 44.85 r = −0.35	307.000; ***p* =< 0.001** MR Group 3 45.73 MR Group 4 25.37 r = −0.45
Difficult to access water	45.199 ***p* =< 0.001**	1062.000; *p* = 0.430 MR Group 1 81.50 MR Group 2 74.11 r = −0.10	411.000; *p* = 0.610 MR Group 1 35.67 MR Group 3 33.39 r = −0.06	73.000; ***p* =< 0.001** MR Group 1 32.44 MR Group 4 16.70 r = −0.61	3138.500; *p* = 0.792 MR Group 2 89.96 MR Group 3 91.95 r = −0.02	494.500; ***p* =< 0.001** MR Group 2 89.23 MR Group 4 32.31 r = −0.51	198.500; ***p* =< 0.001** MR Group 3 47.95 MR Group 4 21.35 r = −0.61
Limited specialised beach mobility equipment	649.238 ***p* =< 0.001**	742.000; ***p* = 0.004** MR Group 1 99.28 MR Group 2 71.66 r = −0.23	315.000; ***p* = 0.027** MR Group 1 41.00 MR Group 3 31.43 r = −0.27	22.000; ***p* =< 0.001** MR Group 1 35.28 MR Group 4 14.81 r = −0.80	3056.500; *p* = 0.584 MR Group 2 89.33 MR Group 3 93.62 r = −0.04	535.000; ***p* =< 0.001** MR Group 2 88.92 MR Group 4 33.81 r = −0.48	220.000; ***p* =< 0.001** MR Group 3 47.51 MR Group 4 22.15 r = −0.58
No hoist availability	12.507 ***p* = 0.006**	880.000; *p* = 0.072 MR Group 1 91.61 MR Group 2 72.72 r = −0.22	284.500; ***p* = 0.023** MR Group 1 42.69 MR Group 3 30.81 r = −0.28	113.500; ***p* = 0.002** MR Group 1 30.19 MR Group 4 18.20 r = −0.46	2793.000; *p* = 0.167 MR Group 2 93.68 MR Group 3 82.00 r = −0.10	2453.500; ***p* = 0.023** MR Group 2 83.69 MR Group 4 59.15 r = −0.21	549.000; *p* = 0.208 MR Group 3 40.80 MR Group 4 34.33 r = −0.14
Limited retail services within 150 m	7.176 *p* = 0.066						
Inaccessible shaded recreational areas	14.786 ***p* = 0.002**	1144.500; *p* = 0.835 MR Group 1 73.08 MR Group 2 75.26 r = −0.03	345.500; *p* = 0.166 MR Group 1 39.31 MR Group 3 32.05 r = −0.17	148.000; ***p* = 0.023** MR Group 1 28.28 MR Group 4 19.48 r = −0.34	2471.000; ***p* = 0.015** MR Group 2 96.14 MR Group 3 75.43 r = −0.18	1066.000; ***p* =< 0.001** MR Group 2 84.86 MR Group 4 53.48 r = −0.27	548.00; *p* = 0.206 MR Group 3 40.82 MR Group 4 34.30 r = −0.15
Lack of physical assistance for transfers	13.523 ***p* = 0.004**	1094.500; *p* = 0.613 MR Group 1 79.69 MR Group 2 74.35 r = −0.06	396.000; *p* = 0.514 MR Group 1 36.50 MR Group 3 33.08 r = −0.08	128.500; ***p* = 0.006** MR Group 1 29.36 MR Group 4 18.76 r = −0.41	3085.500; *p* = 0.683 MR Group 2 91.45 MR Group 3 87.97 r = −0.03	997.500; ***p* =< 0.001** MR Group 2 85.39 MR Group 4 76.91 r = −0.29	431.000; ***p* = 0.010** MR Group 3 43.20 MR Group 4 29.96 r = −0.29
Lack of physical assistance available for beach activities	18.937 ***p* =< 0.001**	1137.000; *p* = 0.801 MR Group 1 77.33 MR Group 2 74.68 r = −0.03	405.000; *p* = 0.600 MR Group 1 36.00 MR Group 3 33.08 r = −0.06	125.500; ***p* = 0.005** MR Group 1 29.53 MR Group 4 18.65 r = −0.42	3057.000; *p* = 0.614 MR Group 2 91.66 MR Group 3 87.39 r = −0.04	840.500; ***p* =< 0.001** MR Group 2 95.58 MR Group 4 50.94 r = −0.35	376.000; ***p* = 0.002** MR Group 3 44.33 MR Group 4 27.93 r = −0.39
Personal safety—fear of falling	16.063 ***p* = 0.001**	1007.000; *p* = 0.302 MR Group 1 65.44 MR Group 2 76.31 r = −0.13	365.000; *p* = 0.217 MR Group 1 39.31 MR Group 3 35.72 r = −0.15	173.500; *p* = 0.099 MR Group 1 26.86 MR Group 4 20.43 r = −0.25	3046.000; *p* = 0.588 MR Group 2 89.25 MR Group 3 93.84 r = −0.04	964.500; ***p* =< 0.001** MR Group 2 85.64 MR Group 4 49.72 r = −0.30	357.000; ***p* =< 0.001** MR Group 3 44.71 MR Group 4 27.22 r = −0.39
Personal safety—reduced confidence outdoor mobility	17.423 ***p* =< 0.001**	927.000; *p* = 0.128 MR Group 1 61.00 MR Group 2 76.92 r = −0.19	351.000; *p* = 0.190 MR Group 1 29.00 MR Group 3 35.84 r = −0.16	189.500; *p* = 0.204 MR Group 1 25.97 MR Group 4 21.02 r = −0.19	3145.500; *p* = 0.831 MR Group 2 90.99 MR Group 3 89.19 r = −0.02	936.000; ***p* =< 0.001** MR Group 2 85.85 MR Group 4 48.67 r = −0.31	355.000; ***p* =< 0.001** MR Group 3 44.76 MR Group 4 27.15 r = −0.39
Personal safety—fear of water	3.813 *p* = 0.282						

Note: MR = mean rank. Group 1 (respondents aged <18 years who identify as having a disability); Group 2 (respondents aged 18–64 years who identify as having a disability); Group 3 (respondents aged ≥65 years who identify as having a disability); Group 4 (respondents aged ≥65 years who do not identify as having a disability). Cell shading is used to highlight the effect size in the Mann–Whitney U test where significant between-group differences were found (white = no significant difference; light blue = significant difference with small effect size (r =< 0.3); mid blue = significant difference with moderate effect size (r = 0.3–<0.5); dark blue = significant difference with large effect size (r =≥ 0.5)).

**Table 3:** Kruskal–Wallis H and Mann–Whitney U results for facilitators to beach access for age and disability status subgroups.

Facilitator	Kruskal−Wallis H	Mann–Whitney U					
		Group 1 vs. Group 2	Group 1 vs. Group 3	Group 1 vs. Group 4	Group 2 vs. Group 3	Group 2 vs. Group 4	Group 3 vs. Group 4
More accessible parking	11.953 ***p* =< 0.008**	1030.000; *p* = < 0.316 MR Group 1 66.72 MR Group 2 76.14 r = −0.08	416.000; *p* = 0.692 MR Group 1 32.61 MR Group 3 34.51 r = −0.05	165.00; *p* = 0.056 MR Group 1 27.33 MR Group 4 20.11 r = −0.28	3026.000; *p* = 0.495 MR Group 2 91.90 MR Group 3 86.76 r = −0.05	1118.000; ***p* = <0.001** MR Group 2 84.47 MR Group 4 55.41 r = −0.27	460.000; ***p* = 0.018** MR Group 3 42.61 MR Group 4 31.04 r = −0.27
Accessible parking closer to beach access point	13.094 ***p* =< 0.004**	1043.500; *p* =< 0.356 MR Group 1 82.53 MR Group 2 73.97 r = −0.08	362.500; *p* = 0.201 MR Group 1 38.36 MR Group 3 32.40 r = −0.16	133.000; ***p* = 0.006** MR Group 1 29.11 MR Group 4 18.93 r = −0.41	2986.500; *p* = 0.411 MR Group 2 92.20 MR Group 3 85.95 r = −0.06	1122.000; ***p* =< 0.001** MR Group 2 84.44 MR Group 4 55.56 r = −0.27	472.000; ***p* = 0.027** MR Group 3 42.37 MR Group 4 31.48 r = −0.25
Accessible lead-up pathways to beach	30.086 ***p* =< 0.001**	924.500; ***p* =< 0.047** MR Group 1 89.14 MR Group 2 73.06 r = −0.16	347.000; *p* = 0.060 MR Group 1 39.22 MR Group 3 32.08 r = −0.23	88.000; ***p* =< 0.001** MR Group 1 31.61 MR Group 4 17.26 r = −0.60	3194.000; *p* = 0.949 MR Group 2 90.38 MR Group 3 90.82 r = −0.00	921.500; ***p* =< 0.001** MR Group 2 85.97 MR Group 4 48.13 r = −0.37	341.500; ***p* = <0.001** MR Group 3 45.03 MR Group 4 26.65 r = −0.45
Sand walkway/access mat	56.402 ***p* < 0.001**	936.000; ***p* =< 0.035** MR Group 1 88.50 MR Group 2 73.15 r = −0.17	360.000; p = 0.053 MR Group 1 38.50 MR Group 3 32.35 r = −0.24	54.000; ***p* =< 0.001** MR Group 1 33.50 MR Group 4 16.00 r = −0.72	3152.500; p = 0.793 MR Group 2 90.06 MR Group 3 91.66 r = −0.02	624.000; ***p* =< 0.001** MR Group 2 88.24 MR Group 4 37.11 r = −0.52	229.500; ***p* =< 0.001** MR Group 3 47.32 MR Group 4 22.50 r = −0.61
Push beach wheeled walker	21.686 ***p* < 0.001**	898.500; ***p* =< 0.081** MR Group 1 59.42 MR Group 2 77.14 r = −0.14	278.500; ***p* = 0.011** MR Group 1 24.97 MR Group 3 37.32 r = −0.31	214.000; *p* = 0.481 MR Group 1 24.61 MR Group 4 21.93 r = −0.11	2712.500; *p* = 0.080 MR Group 2 86.71 MR Group 3 100.64 r = −0.13	1034.500; ***p* =< 0.001** MR Group 2 85.10 MR Group 4 52.31 r = −0.28	292.500; ***p* =< 0.001** MR Group 3 46.03 MR Group 4 24.83 r = −0.49
Wheelchair—sand access	35.981 ***p* < 0.001**	822.000; ***p* =< 0.015** MR Group 1 94.83 MR Group 2 72.27 r = −0.20	248.000; ***p* = 0.002** MR Group 1 44.72 MR Group 3 30.06 r = −0.38	36.000; ***p* =< 0.001** MR Group 1 34.50 MR Group 4 15.33 r = −0.75	2767.000; *p* = 0.111 MR Group 2 93.88 MR Group 3 81.47 r = −0.12	769.500; ***p* =< 0.001** MR Group 2 87.13 MR Group 4 42.50 r = −0.40	376.500; ***p* = 0.001** MR Group 3 44.32 MR Group 4 27.94 r = −0.37
Wheelchair–water access	26.900 ***p* < 0.001**	1083.500; *p* =< 0.528 MR Group 1 80.31 MR Group 2 74.27 r = −0.05	330.500; *p* = 0.091 MR Group 1 40.14 MR Group 3 31.74 r = −0.21	72.000; ***p* = <0.001** MR Group 1 32.50 MR Group 4 16.67 r = −0.62	2725.500; *p* = 0.084 MR Group 2 94.19 MR Group 3 80.62 r = −0.13	798.500; ***p* = <0.001** MR Group 2 86.90 MR Group 4 43.57 r = −0.39	404.500; ***p* = 0.004** MR Group 3 43.74 MR Group 4 28.98 r = −0.33
Accessible toilet	22.042 ***p* < 0.001**	1022.500; *p* =< 0.290 MR Group 1 83.69 MR Group 2 73.81 r = −0.09	307.500; ***p* = 0.038** MR Group 1 41.42 MR Group 3 31.28 r = −0.25	101.500; ***p* = <0.001** MR Group 1 30.86 MR Group 4 17.76 r = −0.52	2693.500; *p* = 0.063 MR Group 2 94.44 MR Group 3 79.97 r = −0.14	953.000; ***p* =< 0.001** MR Group 2 85.73 MR Group 4 49.30 r = −0.33	431.500; ***p* = 0.009** MR Group 3 43.19 MR Group 4 29.98 r = −0.30
Accessible shower	27.454 ***p* < 0.001**	837.000; *p* =< 0.028 MR Group 1 94.00 MR Group 2 72.39 r = −0.18	281.000; ***p* = 0.013** MR Group 1 42.89 MR Group 3 30.73 r = −0.30	62.500; ***p* =< 0.001** MR Group 1 33.03 MR Group 4 16.31 r = −0.65	2986.500; *p* = 0.438 MR Group 2 92.20 MR Group 3 85.95 r = −0.06	868.000; ***p* =< 0.001** MR Group 2 86.37 MR Group 4 46.15 r = −0.35	362.500; ***p* =< 0.001** MR Group 3 44.60 MR Group 4 27.43 r = −0.39
Accessible change room	31.637 ***p* < 0.001**	738.000; ***p* =< 0.004** MR Group 1 99.50 MR Group 2 71.63 r = −0.24	222.000; ***p* = <0.001** MR Group 1 46.17 MR Group 3 29.53 r = −0.42	46.000; ***p* = <0.001** MR Group 1 33.94 MR Group 4 15.70 r = −0.72	2771.500; *p* = 0.127 MR Group 2 93.84 MR Group 3 81.56 r = −0.11	888.000; ***p* = <0.001** MR Group 2 86.22 MR Group 4 46.89 r = −0.35	422.000; ***p* = 0.007** MR Group 3 43.38 MR Group 4 29.63 r = −0.31
Accessible change table	39.115 ***p* < 0.001**	378.000; ***p* = <0.001** MR Group 1 119.50 MR Group 2 68.89 r = −0.40	924.500; ***p* = 0.047** MR Group 1 54.17 MR Group 3 26.59 r = −0.65	30.000; ***p* = <0.001** MR Group 1 34.83 MR Group 4 15.11 r = −0.78	2398.500; ***p* = 0.007** MR Group 2 96.69 MR Group 3 73.95 r = −0.20	1290.500; ***p* = 0.021** MR Group 2 83.15 MR Group 4 61.80 r = −0.18	661.500; *p* = 1.000 MR Group 3 38.50 MR Group 4 38.50 r = −0.00
Hoist availability	12.556 ***p* =< 0.006**	907.500; *p* =< 0.099 MR Group 1 90.08 MR Group 2 72.93 r = −0.14	78.000; ***p* = 0.007** MR Group 1 44.22 MR Group 3 30.24 r = −0.33	134.000; ***p* = 0.007** MR Group 1 29.06 MR Group 4 18.96 r = −0.40	2512.500; ***p* = 0.020** MR Group 2 95.82 MR Group 3 76.28 r = −0.17	1359.500; ***p* = 0.048** MR Group 2 82.62 MR Group 4 64.35 r = −0.16	643.000; *p* = 0.832 MR Group 3 38.12 MR Group 4 39.19 r = −0.02
Food retail services within 150 m	5.117 *p* =< 0.163						
Accessible shaded recreational areas	16.395 ***p* < 0.001**	1165.500; *p* =< 0.918 MR Group 1 75.92 MR Group 2 74.87 r = −0.01	369.000; *p* = 0.285 MR Group 1 38.00 MR Group 3 32.53 r = −0.13	130.000; ***p* = 0.007** MR Group 1 29.28 MR Group 4 18.81 r = −0.40	2716.000; *p* = 0.092 MR Group 2 94.27 MR Group 3 80.43 r = −0.13	969.500; ***p* = <0.001** MR Group 2 85.60 MR Group 4 49.91 r = −0.31	470.500; ***p* = 0.032** MR Group 3 42.40 MR Group 4 31.43 r = −0.25
Accessible playground	44.201 ***p* < 0.001**	470.500; ***p* = <0.001** MR Group 1 114.36 MR Group 2 69.59 r = −0.35	79.500; ***p* = <0.001** MR Group 1 54.08 MR Group 3 26.62 r = −0.64	57.000; ***p* = <0.001** MR Group 1 33.33 MR Group 4 16.11 r = −0.67	1901.000; ***p* = <0.001** MR Group 2 100.49 MR Group 3 63.80 r = −0.33	1269.000; ***p* = 0.016** MR Group 2 83.31 MR Group 4 61.00 r = −0.19	559.000; *p* = 0.238 MR Group 3 36.41 MR Group 4 42.30 r = −0.14
Physical assistance—car transfers	6.680 *p* =< 0.083						
Physical assistance—transfers to beach mobility equipment	11.264 ***p* =< 0.010**	1047.000; *p* =< 0.426 MR Group 1 82.33 MR Group 2 73.99 r = −0.07	351.000; *p* = 0.188 MR Group 1 39.00 MR Group 3 32.16 r = −0.16	144.000; ***p* = 0.017** MR Group 1 28.50 MR Group 4 19.33 r = −0.36	2848.500; *p* = 0.230 MR Group 2 93.26 MR Group 3 83.13 r = −0.09	1126.000; ***p* = 0.002** MR Group 2 84.40 MR Group 4 55.70 r = −0.24	477.500; ***p* = 0.037** MR Group 3 42.26 MR Group 4 31.69 r = −0.24
Physical assistance—personal care activities	6.691 *p* =< 0.082						
Physical assistance—beach activities	16.452 ***p* < 0.001**	1122.500; *p* =< 0.731 MR Group 1 78.14 MR Group 2 74.57 r = −0.03	372.000; *p* = 0.309 MR Group 1 37.83 MR Group 3 32.59 r = −0.12	122.000; ***p* = 0.004** MR Group 1 29.72 MR Group 4 18.52 r = −0.43	2853.500; *p* = 0.234 MR Group 2 93.22 MR Group 3 83.23 r = −0.09	962.500; ***p* =< 0.001** MR Group 2 85.65 MR Group 4 49.65 r = −0.31	409.500; ***p* = 0.004** MR Group 3 43.64 MR Group 4 29.17 r = −0.33
Physical assistance—ocean activities	22.133 ***p* < 0.001**	1141.500; *p* =< 0.817 MR Group 1 77.08 MR Group 2 74.71 r = −0.02	372.500; *p* = 0.313 MR Group 1 37.81 MR Group 3 32.60 r = −0.12	110.500; ***p* = 0.002** MR Group 1 30.36 MR Group 4 18.09 r = −0.47	2793.500; *p* = 0.160 MR Group 2 93.68 MR Group 3 82.01 r = −0.10	809.500; ***p* = <0.001** MR Group 2 86.82 MR Group 4 43.98 r = −0.37	392.500; ***p* = 0.003** MR Group 3 43.99 MR Group 4 28.54 r = −0.34
Accessible beach events and activities	36.814 ***p* < 0.001**	928.000; *p* =< 0.109 MR Group 1 88.94 MR Group 2 73.08 r = −0.13	287.000; ***p* = 0.020** MR Group 1 42.56 MR Group 3 30.86 r = −0.29	41.000; ***p* =< 0.001** MR Group 1 34.22 MR Group 4 15.52 r = −0.72	2749.000; *p* = 0.112 MR Group 2 94.02 MR Group 3 81.10 r = −0.12	632.000; ***p* =< 0.001** MR Group 2 88.18 MR Group 4 37.41 r = −0.44	337.500; ***p* =< 0.001** MR Group 3 45.11 MR Group 4 26.50 r = −0.42
Booking system for accessibility equipment	35.851 ***p* < 0.001**	10155.500; *p*=< 0.283 MR Group 1 84.08 MR Group 2 73.75 r = −0.09	298.500; ***p* = 0.031** MR Group 1 41.92 MR Group 3 31.09 r = −0.26	44.500; ***p* = <0.001** MR Group 1 34.03 MR Group 4 15.65 r = −0.71	2712.500; *p* = 0.084 MR Group 2 94.29 MR Group 3 80.36 r = −0.13	657.500; ***p* = <0.001** MR Group 2 87.98 MR Group 4 38.35 r = −0.43	311.500; ***p* = <0.001** MR Group 3 45.64 MR Group 4 25.54 r = −0.46
Calendar of equipment availability and accessible events/activities	41.084 ***p* < 0.001**	1080.000; *p* =< 0.504 MR Group 1 80.50 MR Group 2 74.24 r = −0.05	369.500; *p* = 0.250 MR Group 1 37.97 MR Group 3 32.54 r = −0.14	47.500; ***p* = <0.001** MR Group 1 33.86 MR Group 4 15.76 r = −0.70	2987.500; *p* = 0.415 MR Group 2 92.19 MR Group 3 85.97 r = −0.06	560.500; ***p* = <0.001** MR Group 2 88.72 MR Group 4 34.76 r = −0.48	250.000; ***p* = <0.001** MR Group 3 46.90 MR Group 4 23.26 r = −0.54

Note: MR = mean rank. Group 1 (respondents aged <18 years who identify as having a disability); Group 2 (respondents aged 18–64 years who identify as having a disability); Group 3 (respondents aged ≥65 years who identify as having a disability); Group 4 (respondents aged ≥65 years who do not identify as having a disability). Cell shading is used to highlight the effect size in the Mann–Whitney U test where significant between-group differences were found (white = no significant difference; light blue = significant difference with small effect size (r =< 0.3); mid blue = significant difference with moderate effect size (r = 0.3–<0.5); dark blue = significant difference with large effect size (r =≥ 0.5)).

## Data Availability

The data presented in this study are available on request from the corresponding author. The data are not publicly available due to privacy and ethical considerations.

## Supplementary Materials

The following supporting information can be downloaded at: https://www.mdpi.com/article/10.3390/ijerph20095651/s1, Supplementary Material S1: 39-item survey questionnaire, barrier and facilitator comparisons for mobility and non-mobility disability subgroups. Supplementary Material S2: Mann-Whitney U results for barriers to beach access for disability and mobility status subgroups. Supplementary Material S3: Mann-Whitney U results for facilitators to beach access for disability and mobility status subgroups.
