# Supplementary material for: Tides of Change—Barriers and Facilitators to Beach Accessibility for Older People and People with Disability: An Australian Community Survey
Source: Int J Environ Res Public Health. 2023 Apr 26;20(9):5651. doi: 10.3390/ijerph20095651 (PMC10178453; doi:10.3390/ijerph20095651)
Supplement: Supplementary file 1 [file ijerph-20-05651-s001.zip › Supplementary Material S1.pdf]

Beach Accessibility - Patient Perspective National Survey 2021

Participant Information Statement

Project Title: Perspectives on the benefits, barriers, and facilitators of beach accessibility in community dwelling older adults and individuals living with a disability or mobility limitations - an Australian community level survey

HREC Number: 0000022411

Chief Investigators
Sasha Job - Chief Investigator, Lecturer Physiotherapy, CQUniversity Bundaberg
Dr Steven Obst - Chief Investigator, Senior Lecturer Physiotherapy, CQUniversity Bundaberg
Dr Luke Heales - Chief Investigator, Senior Lecturer Physiotherapy, CQUniversity BundabergDr Maria O'Reilly - Chief Investigator, Senior Lecturer Occupational Therapy, CQUniversity Bundaberg

What is a participant information statement?
This statement tells you about the research project. It explains to you clearly and openly all the information we would like to gather and what we will do with this information. This information is to help you decide whether or not you would like to take part in the research. Please read this information statement carefully. Before you decide whether or not you want to take part in the research, you can ask us any questions you have about the project. You may want to talk about the project with your family, friends, carer, or healthcare worker. It is ok to say no if you do not want to participate in the research.

Important Things to Know
It is your choice whether or not you would like to take part in the research. You do not have to agree to participate if you do not want to. If you complete this survey, you are telling us that you:

- ✓ Understand what you have read
- ✓ Have had a chance to ask any questions and received satisfactory answers
- ✓ Consent to participate in the project

You are invited to complete the survey if you ...

- ✓ Currently access OR want to access patrolled beaches throughout Australia
- ✓ AND meet at least ONE of the following criteria:
  - Older person (aged 65 years or older)
  - Person living with a disability
  - Person living with a mobility restriction (temporary or permanent)

If you are aged <18 years, the survey must be completed by a parent/ guardian acting on your behalf. If you are invited to complete the survey, but are unable to do so on your own, the survey may be completed by:

- ✓ A parent/ guardian or carer acting on your behalf
- ✓ A service provider who regularly interacts with you acting on your behalf

What do we want to find out?

- ✓ Information you provide will assist us in having a better understanding of: ü Your current levels of physical activity
- ✓ Your preferred patrolled beach location(s)
- ✓ Your patterns of patrolled beach use (e.g. when, how long and why you visit a patrolled beach)
- ✓ Challenges you face getting to a patrolled beach and accessing the beach and surrounds
- ✓ Activities you participate in while at a patrolled beach
- ✓ How a visit to a patrolled beach may affect your mood, health, well-being and participation in physical activity
- ✓ How patrolled beach accessibility may be improved

What will we do with the information you provide?
Information you provide will help inform research and planning initiatives for improved accessibility of patrolled beaches throughout Australia.

How will this information benefit you or others?
The benefits of obtaining this information include supporting us to:

- ✓ Break down the barriers that prevent patrolled beach access and patrolled beach activity participation for all people, regardless of abilities
- ✓ Enable people living with a disability or mobility restriction to enjoy our patrolled beaches as an active space for physical activity, recreation, volunteer opportunities and spectatorship
- ✓ Enhance patrolled beach activity participation, improving physical health, mental functioning, happiness, well being, enjoyment and connection of community members
- ✓ Design patrolled beach activities to support flexible options for physical activity and recreation that meet diverse lifestyles, abilities, and interests

Are there any risks to participating in this survey?
The only risk associated with participating in this survey is one of inconvenience in the time taken to complete the survey. It is estimated that this survey will take up to 15 minutes to complete.

How will the information be kept private?
All information will be stored securely for a minimum of 15 years, in accordance with CQUniversity policy. Information will solely be used for the purposes described. No personal information will be recorded, and all surveys are anonymous. Information will not be used in any way that would allow you to be identified, or your survey responses attributed to you. Only chief investigators will have direct access to the survey information. Data may be accessed by auditors, the ethics committee or regulatory authorities. Data may be used as comparative data in future projects or stored in an open access database for secondary analysis. The results of the study may be published.

Do you Require Further Information About the Survey?

If you have any questions or would like further information, please contact: Sasha Job
Physiotherapy Lecturer
CQUniversity Australia, Bundaberg Campus
Phone: +61 07 4150 7702
Email: s.job@cqu.edu.au

Ethics Contact:

The CQUniversity Human Research Ethics Committee (HREC) has approved this study. If you have any concerns and/or complaints about the project, the way it is being conducted or your rights as a research participant, and would like to speak to someone independent of the project, please contact the HREC Coordinator via email (ethics@cqu.edu.au).

Eligibility Screening Questions

pre1 Do you currently access OR want to access patrolled beaches?

- ☐ Yes
- ☐ No

If no is selected, you are not eligible to complete this survey.

pre2 Do you identify as:
Please select all that apply

- ☐ Older person (65 years or older)
- ☐ Person with a disability
- ☐ Person with a mobility restriction
- ☐ Parent/ guardian or carer acting on behalf of a person with a disability
- ☐ Service provider acting on behalf of a person with a disability
- ☐ None of the above

If none of the above is selected, you are not eligible to complete this survey.

pre3 Are you aged 18 years or older?

- ☐ Yes
- ☐ No

If yes is selected, please ask a parent/ guardian to answer on your behalf.

General Instructions to Complete Survey
Please read each of the questions and select the appropriate answer(s) or provide information as required. If you are completing the survey on behalf of a person with a disability or mobility restriction, please ensure each question applies to their situation or circumstance.

Section 1: Your Background
First, a little bit about you. The following questions aim to help us understand the diversity of our community.

Q1 What is your current age in years? ______________________

Q2 What is your gender?
- ☐ Male
- ☐ Female
- ☐ Other
- ☐ Prefer not to say

Q3 Do you identify as a person of Aboriginal or Torres Strait Islander descent?
- ☐ Yes
- ☐ No
- ☐ Prefer not to say

Q4 Do you identify as a person from a non-English speaking background?
- ☐ Yes
- ☐ No

Q5 What is the postcode of your primary residence?
Please specify: ______________________

Q6 In general, how would you describe your health?
- ☐ Excellent
- ☐ Very good
- ☐ Good
- ☐ Fair
- ☐ Poor

Q7 What is your approximate height in centimetres?
- ☐ Height (centimetres) ______________________
- ☐ Unsure
- ☐ Prefer not to say

If unsure is selected, please answer Q8, otherwise please continue to Q9.

Q8 If you know your approximate height in another measurement unit, please enter below.
- ☐ Measurement unit (e.g. feet and inches) ______________________
- ☐ Height ______________________
- ☐ Unsure

Q9 What is your approximate weight in kilograms?

- ☐ Weight (kilograms) ______________________________
- ☐ Unsure
- ☐ Prefer not to say

If unsure is selected, please answer Q10, otherwise please continue to Q11.

Q10 If you know your approximate weight in another measurement unit, please enter below.

- ☐ Measurement unit (e.g. stones and pounds) ____________________
- ☐ Weight ______________________________
- ☐ Unsure

Q11 Please indicate the level of assistance you require for self care and body movement activities.

	Yes, always	Yes, sometimes	No
Do you require assistance from someone else for self care activities? (e.g. everyday activities such as eating, showering, dressing, toileting)	○	○	○
Do you require assistance from someone else for body movement activities? (e.g. getting out of bed, moving around your home, moving around places in the community)	○	○	○
Do you require a mobility device for walking or moving around your home?	○	○	○
Do you require a mobility device for walking or moving around places in the community?	○	○	○

Q12 Please select the mobility device(s) that you use.
Please select all that apply

- ☐ Walking stick (cane)
- ☐ Crutches
- ☐ Walking frame - pickup
- ☐ Walking frame - wheeled
- ☐ Wheelchair - manual (non-motorised)
- ☐ Wheelchair - motorised
- ☐ Mobility scooter
- ☐ Other - please specify: ______________________________

Section 2: Physical Activity
The following questions aim to help us understand your current level of leisure time physical activity. The questions relate specifically to ***physical activity you participate in solely for recreation, sport, exercise or leisure***. Please do not include any activities related to work, housework, house maintenance or caring for family.

Q13 In a typical week (7 day period), on how many days do you participate in physical activity for recreation, sport, exercise or leisure?

- ☐ Never
- ☐ 1-2 days per week
- ☐ 3-4 days per week
- ☐ 5-6 days per week
- ☐ Every day

If never is selected, please skip to Q18.

Q14 In a typical week (7 day period), on how many days do you participate in ***moderate intensity*** physical activity for at least 10 minutes? Moderate intensity physical activity requires some effort, but still allows you to speak easily while undertaking the activity.

- ☐ Never
- ☐ 1-2 days
- ☐ 3-4 days
- ☐ 5-6 days
- ☐ Every day

Q15 How much time do you usually spend on one of those days participating in moderate intensity physical activity?

- ☐ 10 minutes
- ☐ 11 minutes - ½ hour
- ☐ ½ - 1 hour
- ☐ 1 - 1 ½ hours
- ☐ 1 ½ -2 hours
- ☐ More than 2 hours

Q16 In a typical week (7 day period), on how many days do you participate in ***vigorous intensity*** physical activity for at least 10 minutes?
Vigorous intensity physical activity requires large effort and makes you breathe harder and faster - "huff and puff"

- ☐ Never
- ☐ 1-2 days
- ☐ 3-4 days
- ☐ 5-6 days
- ☐ Every day

Q17 How much time do you usually spend on one of those days participating in vigorous intensity physical activity?

- ☐ 10 minutes
- ☐ 11 minutes - ½ hour
- ☐ ½ - 1 hour
- ☐ 1 - 1 ½ hours
- ☐ 1 ½ -2 hours
- ☐ More than 2 hours

Q18 To what extent do you agree or disagree with the following statement regarding physical activity enjoyment?

	Strongly Agree	Agree	Neither Agree nor Disagree	Disagree	Strongly Disagree
I enjoy doing physical activity	○	○	○	○	○

Q19 A number of situations are described below that can make it hard to stick to a physical activity routine. Please rate how certain you are that you can get yourself to perform your physical activity routine regularly (on most days of the week) from 0 (I cannot do this activity at all) to 10 (I am certain I can do this activity successfully).

Please continue to the next page to answer.

	I cannot do this activity at all										I am certain I can do this activity successfully
	0	1	2	3	4	5	6	7	8	9	10
When I am feeling tired	○	○	○	○	○	○	○	○	○	○	○
When I am feeling under pressure from work	○	○	○	○	○	○	○	○	○	○	○
During bad weather	○	○	○	○	○	○	○	○	○	○	○
After recovering from an injury that caused me to stop exercising	○	○	○	○	○	○	○	○	○	○	○
During or after experiencing personal problems	○	○	○	○	○	○	○	○	○	○	○
When I am feeling depressed	○	○	○	○	○	○	○	○	○	○	○
When I am feeling anxious	○	○	○	○	○	○	○	○	○	○	○
After recovering from an illness that caused me to stop exercising	○	○	○	○	○	○	○	○	○	○	○
When I feel physical discomfort when I exercise	○	○	○	○	○	○	○	○	○	○	○
After a vacation	○	○	○	○	○	○	○	○	○	○	○
When I have too much work to do at home	○	○	○	○	○	○	○	○	○	○	○
When visitors are present	○	○	○	○	○	○	○	○	○	○	○
When there are other interesting things to do	○	○	○	○	○	○	○	○	○	○	○
If I don't reach my exercise goals	○	○	○	○	○	○	○	○	○	○	○
Without support from my family or friends	○	○	○	○	○	○	○	○	○	○	○
During a vacation	○	○	○	○	○	○	○	○	○	○	○
When I have other time commitments	○	○	○	○	○	○	○	○	○	○	○
After experiencing family problems	○	○	○	○	○	○	○	○	○	○	○

Section 3: Beach Preferences
The following questions aim to help us understand where, when, and why you visit the beach, as well as benefits of visiting the beach.

Q20 How far is the closest ***patrolled beach*** from your primary residence?

- ☐ 0-2km
- ☐ 3-10km
- ☐ 11-20km
- ☐ 21-30km
- ☐ More than 30km

Q21 Which ***patrolled beaches*** do you visit most often?
Please order the patrolled beaches from the beach you visit most often (1) to the beach you visit least often (5).

- ☐ I am currently unable to visit the beach due to access limitations (i.e. I have
- ☐ difficulty travelling to the beach and/or accessing the beach and surrounds)
- ☐ (1) Please specify: ______________________________
- ☐ (2) Please specify: ______________________________
- ☐ (3) Please specify: ______________________________
- ☐ (4) Please specify: ______________________________
- ☐ (5) Please specify: ______________________________

*If you cannot currently access the beach, please proceed with this section of the survey by answering questions marked only in **blue** or **black** (21a, 22, 26, 27a, 28a, 29a, 31).*

*If you can currently access the beach, please proceed with this section of the survey by answering questions marked only in **purple** or **black** (22, 23, 24, 25, 26, 27. 28, 29, 30. 31).*

Q21a If you were able to visit the beach, which ***patrolled beaches*** *would* you visit most often?
Please order the patrolled beaches from the beach you *would* visit most often (1) to the beach you *would* visit least often (5).

- ☐ (1) Please specify: ______________________________
- ☐ (2) Please specify: ______________________________
- ☐ (3) Please specify: ______________________________
- ☐ (4) Please specify: ______________________________
- ☐ (5) Please specify: ______________________________

Q22 To what extent do you agree or disagree that the following would positively influence your selection of which beach to visit (any beach, any location)?
Everyone is to complete this question.

Please continue to the next page to answer.

	Strongly Agree	Agree	Neither Agree nor Disagree	Disagree	Strongly Disagree
Proximity to your primary residence	○	○	○	○	○
Patrolled beach	○	○	○	○	○
Ease of travelling to the beach	○	○	○	○	○
Accessibility of the beach (sand and ocean)	○	○	○	○	○
Accessible facilities (e.g. parking, footpaths, toilets, showers, change room facilities, retail services)	○	○	○	○	○
Accessible recreational facilities (e.g. seating, shade, BBQ facilities, playground)	○	○	○	○	○
High water quality	○	○	○	○	○
Sandy beach	○	○	○	○	○
Wide beach	○	○	○	○	○
Calm water	○	○	○	○	○
Good surf	○	○	○	○	○
No litter	○	○	○	○	○
No overcrowding	○	○	○	○	○
Close to towns	○	○	○	○	○
Nearby accommodation	○	○	○	○	○
Ability to take dogs	○	○	○	○	○
Dogs prohibited	○	○	○	○	○
Opportunity to participate in fishing activities	○	○	○	○	○
Opportunities to view wildlife	○	○	○	○	○
Historical significance (e.g. shipwrecks)	○	○	○	○	○
Presence of dunes	○	○	○	○	○
Presence of cliffs	○	○	○	○	○
Presence of rock platforms	○	○	○	○	○
Presence of rocky shores	○	○	○	○	○
Presence of estuary	○	○	○	○	○
Presence of mudflat	○	○	○	○	○

Q23 How often do you visit the beach (any beach, any location)?
If you are currently unable to visit the beach, please skip this question.

- ☐ 1-2 per year
- ☐ 1-2 per half year (6 months)
- ☐ 1-2 per quarter (3 months)
- ☐ 1-2 per month
- ☐ 1-2 per fortnight
- ☐ 1-2 per week
- ☐ 3-4 per week
- ☐ 5 or more per week

Q24 How much time do you usually spend at the beach each time you visit (any beach, any location)?
If you are currently unable to visit the beach, please skip this question.

- ☐ Under 1/2 hour
- ☐ 1/2 - 1 hour
- ☐ 1 - 2 hours
- ☐ More than 2 hours

Q25 How would you rate your level of physical exertion whilst doing your usual activities when you visit the beach (any beach, any location)?
If you are currently unable to visit the beach, please skip this question.

- ☐ Sedentary activity
- ☐ Light activity
- ☐ Moderate activity
- ☐ Vigorous activity

Q26 To what extent do you agree or disagree with the following statements?
Everyone is to complete this question.

	Strongly Agree	Agree	Neither Agree nor Disagree	Disagree	Strongly Disagree
I am able to visit the beach as often as I would like to	○	○	○	○	○
I am able to spend as much time at the beach as I would like to	○	○	○	○	○
If beach accessibility was improved, I would visit the beach more often	○	○	○	○	○
If beach accessibility was improved, I would spend more time at the beach	○	○	○	○	○
If beach accessibility was improved, it would improve my experience of visiting the beach	○	○	○	○	○

Q27 Which day(s) of the week are you most likely to visit the beach?
Please select all that apply
If you are currently unable to visit the beach, please skip this question.

- ☐ Monday
- ☐ Tuesday
- ☐ Wednesday
- ☐ Thursday
- ☐ Friday
- ☐ Saturday
- ☐ Sunday
- ☐ No preference

Q27a If you were able to visit the beach, which day(s) of the week *would* you be most likely to visit the beach?
Please select all that apply
If you are currently able to visit the beach, please skip this question.

- ☐ Monday
- ☐ Tuesday
- ☐ Wednesday
- ☐ Thursday
- ☐ Friday
- ☐ Saturday
- ☐ Sunday
- ☐ No preference

Q28 Which season(s) are you most likely to visit the beach?
Please select all that apply
If you are currently unable to visit the beach, please skip this question.

- ☐ Summer (December, January, February)
- ☐ Autumn (March, April, May)
- ☐ Winter (June, July, August)
- ☐ Spring (September, October, November)
- ☐ No preference

Q28a If you were able to visit the beach, which season(s) *would* you be most likely to visit the beach?
Please select all that apply
If you are currently able to visit the beach, please skip this question.

- ☐ Summer (December, January, February)
- ☐ Autumn (March, April, May)
- ☐ Winter (June, July, August)
- ☐ Spring (September, October, November)
- ☐ No preference

Q29 Why do you visit the beach (any beach, any location)?
Please select all that apply
If you are currently unable to visit the beach, please skip this question.

- ☐ Meet friends or family
- ☐ Build sandcastles
- ☐ Collect shells
- ☐ Read
- ☐ Sunbake
- ☐ Relax/meditate
- ☐ Supervise children
- ☐ Games
- ☐ Picnic
- ☐ Dine out/visit cafes
- ☐ Walk
- ☐ Dog walk/exercise
- ☐ Hike
- ☐ Run
- ☐ Swim
- ☐ Snorkel
- ☐ Bodyboard
- ☐ Dive
- ☐ Kayak
- ☐ Surf
- ☐ Fish
- ☐ Horse ride
- ☐ Cycle
- ☐ Surf lifesaving club activities
- ☐ Rockpool-ramble
- ☐ Sight-see
- ☐ Photography
- ☐ Observe wildlife
- ☐ Observe birds
- ☐ Camp
- ☐ Water/jet ski
- ☐ Dune surf
- ☐ 4WD/off road vehicle driving
- Other

Q29a Why do you visit the beach (any beach, any location)?
Please select all that apply
If you are currently able to visit the beach, please skip this question.

- ☐ Meet friends or family
- ☐ Build sandcastles
- ☐ Collect shells
- ☐ Read
- ☐ Sunbake
- ☐ Relax/meditate
- ☐ Supervise children
- ☐ Games
- ☐ Picnic
- ☐ Dine out/visit cafes
- ☐ Walk
- ☐ Dog walk/exercise
- ☐ Hike
- ☐ Run
- ☐ Swim
- ☐ Snorkel
- ☐ Bodyboard
- ☐ Dive
- ☐ Kayak
- ☐ Surf
- ☐ Fish
- ☐ Horse ride
- ☐ Cycle
- ☐ Surf lifesaving club activities
- ☐ Rockpool-ramble
- ☐ Sight-see
- ☐ Photography
- ☐ Observe wildlife
- ☐ Observe birds
- ☐ Camp
- ☐ Water/jet ski
- ☐ Dune surf
- ☐ 4WD/off road vehicle driving
- Other

Q30 To what extent do you agree or disagree that the following are benefits for you when you visit the beach (any beach, any location)?
If you are currently unable to visit the beach, please skip this question.

	Strongly Agree	Agree	Neither Agree nor Disagree	Disagree	Strongly Disagree
Physical benefits such as increased fitness, functioning, muscle strength, flexibility	○	○	○	○	○
Decreased stress and tension	○	○	○	○	○
Revitalisation and increased energy	○	○	○	○	○
Enjoyment and increased wellbeing	○	○	○	○	○
Meeting and or connecting with others	○	○	○	○	○
Entertainment or fun	○	○	○	○	○
Regular involvement in structured group activities	○	○	○	○	○
Casual participation in informal activities	○	○	○	○	○
Opportunity to challenge abilities and/or achieve goals	○	○	○	○	○
Time to yourself	○	○	○	○	○

Q31
If your ability to visit or use patrolled beaches is restricted, please tell us:
- ***Why*** are you unable to access your preferred patrolled beach(es)?
- ***What*** would improve your ability to visit your preferred patrolled beach(es)?

Section 4: Beach Accessibility

The following questions aim to help us understand how accessible our ***patrolled beaches*** currently are and how accessibility may be improved. An accessible beach ensures that anyone can enjoy the beach. There is consideration of ***travelling to the beach*** (e.g. transport and infrastructure) as well as ***accessing the beach*** (soft sand, hard sand and ocean) ***and surrounds*** (e.g. dunes, grassed areas, recreational facilities).

Q32 To what extent do you agree or disagree with the following statements regarding your current ability to visit your preferred **patrolled beach**? *Please note: this question refers to both travelling to the beach, and accessing the beach (soft sand, hard sand, ocean) and surrounds (e.g. dunes, grassed areas, recreational facilities)*

	It is easy to travel to the beach					It is easy to access the beach and surrounds				
	Strongly Agree	Agree	Neither Agree nor Disagree	Disagree	Strongly Disagree	Strongly Agree	Agree	Neither Agree nor Disagree	Disagree	Strongly Disagree
My preferred patrolled beach	O	O	O	O	O	O	O	O	O	O

Q33 To what extent do you agree or disagree that the following are challenges for you to travel to your preferred patrolled beach from your primary residence.

	Strongly Agree	Agree	Neither Agree nor Disagree	Disagree	Strongly Disagree
Personal health status	O	O	O	O	O
Fatigue	O	O	O	O	O
Financial costs	O	O	O	O	O
Distance from your primary residence	O	O	O	O	O
Limited public transport options	O	O	O	O	O
Limited private transport options	O	O	O	O	O
Limited personal assistance options	O	O	O	O	O
Traffic safety	O	O	O	O	O
Inappropriate, inaccessible, or lack of street crossings	O	O	O	O	O
Inaccessible curbs	O	O	O	O	O
Inappropriate, inaccessible, or lack of footpaths	O	O	O	O	O
Inappropriate, inaccessible, or lack of ramps	O	O	O	O	O
Poor lighting (e.g. roads, footpaths)	O	O	O	O	O

Q34 To what extent do you agree or disagree that the following are challenges for you to access your preferred patrolled beach and surrounds?

Please turn to the next page to answer.

	Strongly Agree	Agree	Neither Agree nor Disagree	Disagree	Strongly Disagree
Insufficient accessible parking	○	○	○	○	○
Parking proximity to beach access point	○	○	○	○	○
Physically challenging geographical features in areas leading to beach (e.g. hills, slippery surfaces, grass, rocks)	○	○	○	○	○
Inappropriate, inaccessible, or lack of leadup pathways and ramps to the beach	○	○	○	○	○
Inappropriate, inaccessible, or lack of toilet	○	○	○	○	○
Inappropriate, inaccessible, or lack of shower	○	○	○	○	○
Inappropriate, inaccessible, or lack of change room facilities	○	○	○	○	○
Difficulty moving on soft sand	○	○	○	○	○
Difficulty moving on hard sand	○	○	○	○	○
Difficult to access the water	○	○	○	○	○
No or limited speacialised beach mobility equipment	○	○	○	○	○
No hoist availability	○	○	○	○	○
No or limited retail services within 150m	○	○	○	○	○
Inappropriate, inaccessible, or lack of shaded recreational areas (tables, chairs, BBQ facilities)	○	○	○	○	○
Lack of physical assistance available for transfers	○	○	○	○	○
Lack of physical assistance available for beach activities	○	○	○	○	○
Personal safety concerns - fear of falling	○	○	○	○	○
Personal safety concerns - reduced confidence with outdoor mobility	○	○	○	○	○
Personal safety concerns - fear of water	○	○	○	○	○

Q35 To what extent do you agree or disagree that the following would improve your ability to visit your preferred patrolled beach, or improve your experience once there?

	Strongly Agree	Agree	Neither Agree nor Disagree	Disagree	Strongly Disagree
More accessible parking	○	○	○	○	○
Accessible parking located closer to beach access point	○	○	○	○	○
Accessible leadup pathways and ramps to the beach	○	○	○	○	○
Sand walkway/ access mat (for pedestrians, wheelchair users, prams, and light ATVs) 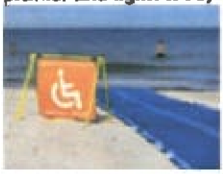	○	○	○	○	○
Push beach wheeled walker 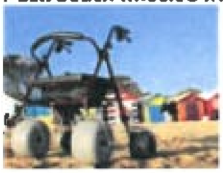	○	○	○	○	○
Wheelchair to enable sand access 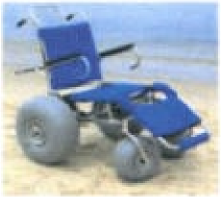	○	○	○	○	○
Wheelchair to enable water access 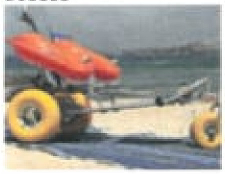	○	○	○	○	○

	Strongly Agree	Agree	Neither Agree nor Disagree	Disagree	Strongly Disagree
Accessible toilet	○	○	○	○	○
Accessible shower	○	○	○	○	○
Accessible change room facilities	○	○	○	○	○
Accessible change table	○	○	○	○	○
Hoist availability	○	○	○	○	○
Food retail services within 150m	○	○	○	○	○
Accessible and shaded recreational areas (tables, chairs, BBQ facilities)	○	○	○	○	○
Accessible playground	○	○	○	○	○
Physical assistance for car transfers	○	○	○	○	○
Physical assistance for transfers to specialised beach equipment	○	○	○	○	○
Physical assistance for personal care activities	○	○	○	○	○
Physical assistance for beach activities	○	○	○	○	○
Physical assistance for water (ocean) activities	○	○	○	○	○
Accessible beach events and activities	○	○	○	○	○
Booking system for accessibility equipment	○	○	○	○	○
Calendar of equipment availability and accessible events and activities	○	○	○	○	○

Q36 Why do you like to visit the beach?

Q37 How has your experience of the beach been affected by your mobility limitations?

Q38 How do you think improving accessibility at patrolled beaches would impact you?

Q39 Is there anything else you would like to tell us regarding your beach access or experiences?

End of Survey

We are very appreciative of the time you have taken to assist us in our research. The information you provided will assist us in future research and planning initiatives for accessible beaches.

If you would like to receive the results of this survey and information on upcoming beach accessibility research, please enter your email address below.
