# Supplementary material for: Tides of Change—Barriers and Facilitators to Beach Accessibility for Older People and People with Disability: An Australian Community Survey
Source: Int J Environ Res Public Health. 2023 Apr 26;20(9):5651. doi: 10.3390/ijerph20095651 (PMC10178453; doi:10.3390/ijerph20095651)
Supplement: Supplementary file 1 [file ijerph-20-05651-s001.zip › Supplementary Material S2.pdf]

Supplementary Material S2. Mann-Whitney U results for barriers to beach access for disability and mobility status subgroups

Barrier	Mann-Whitney U<br>Mobility vs. non-mobility disability	Barrier	Mann-Whitney U<br>Mobility vs. non-mobility disability
Limited accessible parking	2325.500; $p = 0.158$<br>MR Mobility<br>74.02<br>MR Non-mobility<br>84.12<br>r = -0.11	Limited specialised beach mobility equipment	2580.000; $p = 0.683$<br>MR Mobility<br>78.46<br>MR Non-mobility<br>75.68<br>r = -0.03
Parking proximity to beach access point	2625.000; $p = 0.836$<br>MR Mobility<br>76.99<br>MR Non-mobility<br>78.47<br>r = -0.02	No hoist availability	2092.500; **$p = 0.022$**<br>MR Mobility<br>71.72<br>MR Non-mobility<br>88.52<br>r = -0.18
Physically challenging geography leading to beach	2501.000; $p = 0.473$<br>MR Mobility<br>79.24<br>MR Non-mobility<br>74.19<br>r = -0.06	Limited retail services within 150m	2384.000; $p = 0.246$<br>MR Mobility<br>74.60<br>MR Non-mobility<br>83.02<br>r = -0.09
Inaccessible leadup pathways to beach	2628.500; $p = 0.843$<br>MR Mobility<br>77.98<br>MR Non-mobility<br>76.59<br>r = -0.02	Inaccessible shaded recreational areas	2282.500; $p = 0.122$<br>MR Mobility<br>73.60<br>MR Non-mobility<br>84.93<br>r = -0.12
Inaccessible toilet	1891.000; **$p = 0.002$**<br>MR Mobility<br>69.73<br>MR Non-mobility<br>92.31<br>r = -0.25	Lack of physical assistance for transfers	2459.500; $p = 0.398$<br>MR Mobility<br>75.35<br>MR Non-mobility<br>81.59<br>r = -0.07
Inaccessible shower	1866.500; **$p = 0.001$**<br>MR Mobility<br>69.48<br>MR Non-mobility<br>92.78<br>r = -0.26	Lack of physical assistance available for beach activities	2582.500; $p = 0.713$<br>MR Mobility<br>76.57<br>MR Non-mobility<br>79.27<br>r = -0.03
Inaccessible change room	1925.500; **$p = 0.003$**<br>MR Mobility<br>70.06<br>MR Non-mobility<br>91.67<br>r = -0.24	Personal safety – fear of falling	2676.000; $p = 0.998$<br>MR Mobility<br>77.50<br>MR Non-mobility<br>77.49<br>r = -0.00
Difficulty moving – soft sand	2492.000; $p = 0.316$<br>MR Mobility<br>79.33<br>MR Non-mobility<br>74.02<br>r = -0.08	Personal safety – reduced confidence outdoor mobility	2617.000; $p = 0.815$<br>MR Mobility<br>76.91<br>MR Non-mobility<br>78.62<br>r = -0.02
Difficulty moving – hard sand	2473.500; $p = 0.422$<br>MR Mobility<br>75.49	Personal safety – fear of water	2564.000; $p = 0.658$<br>MR Mobility<br>76.39

	MR Non-mobility 81.33 r = -0.06	MR Non-mobility 79.62 r = -0.04
Difficult to access water	2660.500; p=0.944 MR Mobility 77.66 MR Non-mobility 77.20 r=-0.01	

Note: MR = mean rank. Cell shading is used to highlight the effect size in the Mann-Whitney-U test where significant between group differences were found (white = no significant difference; light blue = significant difference with small effect size (r=<0.3); mid blue = significant difference with moderate effect size (r=0.3 – <0.5); dark blue = significant difference with large effect size (r = $\geq$ 0.5)).
