# Supplementary material for: Tides of Change—Barriers and Facilitators to Beach Accessibility for Older People and People with Disability: An Australian Community Survey
Source: Int J Environ Res Public Health. 2023 Apr 26;20(9):5651. doi: 10.3390/ijerph20095651 (PMC10178453; doi:10.3390/ijerph20095651)
Supplement: Supplementary file 1 [file ijerph-20-05651-s001.zip › Supplementary Material S3.pdf]

Supplementary Material S3. Mann-Whitney U results for facilitators to beach access for disability and mobility status subgroups

Facilitator	Mann-Whitney U<br>Mobility vs. non-mobility disability	Facilitator	Mann-Whitney U<br>Mobility vs. non-mobility disability
More accessible parking	2271.500; *p* = 0.078<br>MR Mobility<br>73.49<br>MR Non-mobility<br>85.14<br>r = -0.14	Food retail services within 150m	2463.500; *p* = 0.400<br>MR Mobility<br>79.61<br>MR Non-mobility<br>73.48<br>r = -0.07
Accessible parking closer to beach access point	2556.500; *p* = 0.601<br>MR Mobility<br>76.31<br>MR Non-mobility<br>79.76<br>r = -0.04	Accessible shaded recreational areas	2516.000; *p* = 0.517<br>MR Mobility<br>75.91<br>MR Non-mobility<br>80.53<br>r = -0.05
Accessible leadup pathways to beach	2626.000; *p* = 0.802<br>MR Mobility<br>77.00<br>MR Non-mobility<br>78.45<br>r = -0.02	Accessible playground	2439.500; *p* = 0.349<br>MR Mobility<br>75.15<br>MR Non-mobility<br>81.97<br>r = -0.08
Sand walkway/ access mat	2459.500; *p* = 0.237<br>MR Mobility<br>79.65<br>MR Non-mobility<br>73.41<br>r = -0.10	Physical assistance – car transfers	2654.500; *p* = 0.931<br>MR Mobility<br>77.72<br>MR Non-mobility<br>77.08<br>r = -0.01
Push beach wheeled walker	2454.500; *p* = 0.359<br>MR Mobility<br>79.70<br>MR Non-mobility<br>73.31<br>r = -0.07	Physical assistance – transfers to beach mobility equipment	2624.000; *p* = 0.837<br>MR Mobility<br>78.02<br>MR Non-mobility<br>76.51<br>r = -0.02
Wheelchair – sand access	2313.000; *p* = 0.125<br>MR Mobility<br>73.90<br>MR Non-mobility<br>84.36<br>r = -0.12	Physical assistance – personal care activities	2384.500; *p* = 0.248<br>MR Mobility<br>74.61<br>MR Non-mobility<br>83.01<br>r = -0.09
Wheelchair – water access	2170.000; ***p* = 0.033**<br>MR Mobility<br>72.49<br>MR Non-mobility<br>87.06<br>r = -0.17	Physical assistance – beach activities	2324.000; *p* = 0.163<br>MR Mobility<br>74.01<br>MR Non-mobility<br>84.15<br>r = -0.11
Accessible toilet	2181.500; ***p* = 0.033**<br>MR Mobility<br>72.60<br>MR Non-mobility<br>86.84<br>r = -0.17	Physical assistance – ocean activities	2368.000; *p* = 0.218<br>MR Mobility<br>74.45<br>MR Non-mobility<br>83.32<br>r = -0.10

Accessible shower	2261.500; $p = 0.086$<br>MR Mobility<br>73.39<br>MR Non-mobility<br>85.33<br>r = -0.14	Accessible beach events and activities	2514.000; $p = 0.508$<br>MR Mobility<br>75.89<br>MR Non-mobility<br>80.57<br>r = -0.01
Accessible change room	2195.000; ***p* = 0.047**<br>MR Mobility<br>72.73<br>MR Non-mobility<br>86.58<br>r = -0.16	Booking system for accessibility equipment	2337.500; $p = 0.164$<br>MR Mobility<br>74.14<br>MR Non-mobility<br>83.90<br>r = -0.11
Accessible change table	2330.000; $p = 0.170$<br>MR Mobility<br>74.07<br>MR Non-mobility<br>84.04<br>r = -0.11	Calendar of equipment availability and accessible events/ activities	2573.500; $p = 0.654$<br>MR Mobility<br>76.48<br>MR Non-mobility<br>79.44<br>r = -0.04
Hoist availability	2175.500; ***p* = 0.047**<br>MR Mobility<br>72.54<br>MR Non-mobility<br>86.95<br>r = -0.16		

Note: MR = mean rank. Cell shading is used to highlight the effect size in the Mann-Whitney-U test where significant between group differences were found (white = no significant difference; light blue = significant difference with small effect size (r=<0.3); mid blue = significant difference with moderate effect size (r=0.3 – <0.5); dark blue = significant difference with large effect size (r = ≥ 0.5)).
